# The complete chloroplast genome sequence of *Vitis champinii*

**DOI:** 10.1080/23802359.2020.1791005

**Published:** 2020-07-15

**Authors:** Jiang Xiang, Lingzhu Wei, Jianhui Cheng, Mingshan Li, Pengfei Cui, Jiang Wu

**Affiliations:** Institute of Horticulture, Zhejiang Academy of Agricultural Sciences, Hangzhou, China

**Keywords:** *Vitis champinii*, chloroplast genome, rootstock, phylogenetic analysis

## Abstract

*Vitis champinii* is a grapevine rootstock species and widely used in vineyards and in rootstock breeding programs for regions with high nematode populations or saline soils. Here, the complete chloroplast genome of *V. champinii* was reported. The length of the chloroplast genome was 160,657 bp with a large single copy region of 89,217 bp, a small single copy region of 19,504 bp and two separated inverted regions of 51,936 bp, respectively. In total, 130 unique genes were identified of this genome, including 85 protein-coding genes, 37 tRNA genes, and 8 rRNA genes. Phylogenetic analysis indicates that *V. champinii is* closely related to *Vitis acerifolia*.

*Vitis champinii*, which is one of the most important grape rootstock species in the world, has strong resistance to nematodes, phylloxera, and is tolerant of lime and saline soils (Lowe and Walker 2006). In this study, the complete chloroplast genome of *V. champinii* was sequenced for the first time, which will be beneficial to elucidate its characteristics at the organelle level and to promote its utilization in rootstock breeding programs.

The total genomic DNA was extracted from fresh leaves of *V. champinii* using the CTAB method (Qu et al. 1996). The voucher specimen was deposited in the Herbarium of the Institute of Horticulture, Zhejiang Academy of Agricultural Sciences (Accession number: ZAAS-XJ-20-07). Leaf samples were collected from Research Vineyard for Grape Germplasm and Breeding in Zhejiang Academy of Agricultural Sciences, Xucun Town, Haining City, Zhejiang Province, China (120°24′15″E; 30°26′15″N). The genome library (paired-end, PE = 150 bp) was sequenced on an Illumina NovaSeq 6000 platform (Illumina Inc., San Diego, CA, USA). Totally, 11.9 Gb sequence clean reads were obtained and used to assemble the chloroplast genome using NOVOPlasty v3.8.3 (Nicolas et al. 2016). The chloroplast genome of *V. vinifera* (DQ424856) was used as the reference for assembly and annotation. The GeSeq package and BLAST 2.6.0+ were used to predicted and annotate genes, respectively (Michael et al. 2017).

The complete chloroplast genome of *V. champinii* (GenBank:MT604109) was 160,657 bp in length, including a large single copy region (LSC; 89,217 bp), a small single copy region (SSC; 19,504 bp), and a pair of inverted repeats (IRs; 51,936 bp). The genome contained a total of 130 unique genes, including 85 protein-coding genes (PCGs), 37 tRNA genes, and 8 rRNA genes. Among these genes, 6 PCGs, 7 tRNAs, and 4 rRNAs have two copies, and only one tRNA (trnM-CAU) have four copies.

To investigate the phylogenetic position of *V. champinii* within Vitaceae, a neighbour-joining phylogenetic tree was constructed together with 22 other grapevine species by using MEGA X (Kumar et al. 2018). Based on the topology of the phylogenetic tree (Figure 1), all of the studied *Vitis* species separated into *Euvitis* subgenus and *Muscadinia* subgenus. *Vitis champinii* is closely related to *Vitis acerifolia* and both belong to America species clade.

**Figure 1.**
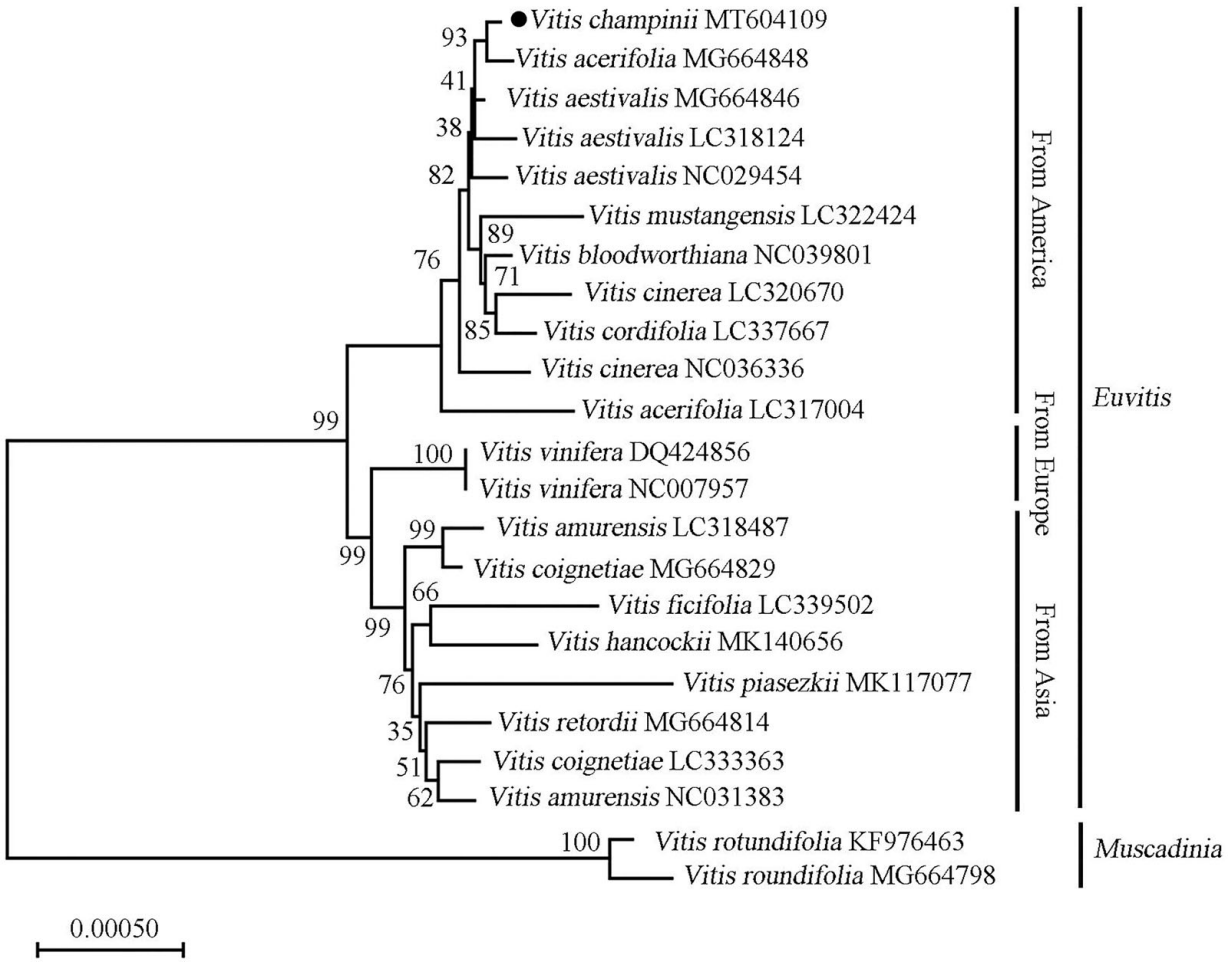
The phylogenetic relationship of 23 species within the Vitis species based on neighbour-joining analysis of chloroplast genomes. The bootstrap values were based on 1000 replicates and were shown next to the nodes.

## Data Availability

The data that support the findings of this study are openly available in Genbank at https://www.ncbi.nlm.nih.gov/genbank/, reference number [MT604109], or available from the corresponding author.
